# Bis(4-fluoro­benzoato-κ^2^ *O*,*O*′)bis­(nicotinamide-κ*N* ^1^)zinc(II) monohydrate

**DOI:** 10.1107/S1600536808003747

**Published:** 2008-02-06

**Authors:** Tuncer Hökelek, Nagihan Çaylak, Hacali Necefoğlu

**Affiliations:** a Department of Physics, Hacettepe University, 06800 Beytepe, Ankara, Turkey; b Department of Physics, Faculty of Arts and Science, Sakarya University, 54187 Esentepe, Adapazarı, Turkey; c Department of Chemistry, Kafkas University, 63100 Kars, Turkey

## Abstract

The title compound, [Zn(C_7_H_4_FO_2_)_2_(C_6_H_6_N_2_O)_2_]·H_2_O, is a monomeric complex. It contains two 4-fluoro­benzoate and two nicotinamide ligands and one uncoordinated water mol­ecule. The 4-fluoro­benzoates act as bidentate chelating ligands, while the nicotinamides are monodentate. The six-coordinate geometry around the Zn^II^ atom may be described as highly distorted octa­hedral, with the two nicotinamide ligands arranged *cis*. Inter­molecular O—H⋯O and N—H⋯O hydrogen bonds link the mol­ecules into a supra­molecular structure.

## Related literature

For general background, see: Adiwidjaja *et al.* (1978 ▶); Amiraslanov *et al.* (1979 ▶); Antsyshkina *et al.* (1980 ▶); Bigoli *et al.* (1972 ▶); Day & Selbin (1969 ▶); Krishnamachari (1974 ▶); Nadzhafov, Shnulin & Mamedov (1981 ▶); Shnulin *et al.* (1981 ▶). For related structures, see: Amiraslanov *et al.* (1980 ▶); Capilla & Aranda (1979 ▶); Clegg *et al.* (1986*a* ▶,*b* ▶, 1987 ▶); Guseinov *et al.* (1984 ▶); Hökelek *et al.* (2007 ▶); Hökelek & Necefoğlu (1996 ▶, 2001 ▶); Nadzhafov, Usubaliev *et al.* (1981 ▶); Necefoğlu *et al.* (2002 ▶); Niekerk *et al.* (1953 ▶); Usubaliev *et al.* (1992 ▶).
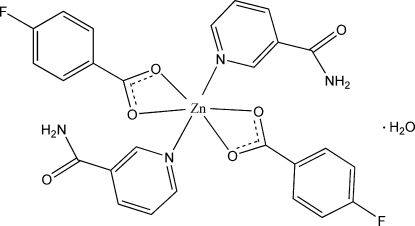


## Experimental

### 

#### Crystal data

[Zn(C_7_H_4_FO_2_)_2_(C_6_H_6_N_2_O)_2_]·H_2_O
*M* *_r_* = 605.87
Triclinic, 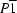

*a* = 8.2363 (2) Å
*b* = 12.3711 (2) Å
*c* = 14.8971 (3) Å
α = 113.178 (14)°
β = 99.015 (17)°
γ = 99.465 (16)°
*V* = 1334.7 (2) Å^3^
*Z* = 2
Mo *K*α radiation
μ = 0.99 mm^−1^
*T* = 294 (2) K
0.30 × 0.25 × 0.20 mm

#### Data collection

Enraf–Nonius TurboCAD-4 diffractometer
Absorption correction: ψ scan (North *et al.*, 1968 ▶) *T*_min_ = 0.735, *T*_max_ = 0.816
5794 measured reflections
5401 independent reflections
4454 reflections with *I* > 2σ(*I*)
*R*_int_ = 0.058
3 standard reflections frequency: 120 min intensity decay: 1%

#### Refinement

*R*[*F* ^2^ > 2σ(*F* ^2^)] = 0.058
*wR*(*F* ^2^) = 0.152
*S* = 1.14
5401 reflections
368 parameters
4 restraints
H atoms treated by a mixture of independent and constrained refinement
Δρ_max_ = 0.70 e Å^−3^
Δρ_min_ = −0.90 e Å^−3^

### 

Data collection: *CAD-4 EXPRESS* (Enraf–Nonius, 1994 ▶); cell refinement: *CAD-4 EXPRESS*; data reduction: *XCAD4* (Harms & Wocadlo, 1995 ▶); program(s) used to solve structure: *SHELXS97* (Sheldrick, 2008 ▶); program(s) used to refine structure: *SHELXL97* (Sheldrick, 2008 ▶); molecular graphics: *ORTEP-3 for Windows* (Farrugia, 1997 ▶); software used to prepare material for publication: *WinGX* (Farrugia, 1999 ▶).

## Supplementary Material

Crystal structure: contains datablocks I, global. DOI: 10.1107/S1600536808003747/hy2118sup1.cif

Structure factors: contains datablocks I. DOI: 10.1107/S1600536808003747/hy2118Isup2.hkl

Additional supplementary materials:  crystallographic information; 3D view; checkCIF report

## Figures and Tables

**Table:** 

Zn—O1	1.978 (2)
Zn—O2	2.564 (3)
Zn—O3	2.010 (3)
Zn—O4	2.458 (3)
Zn—N1	2.079 (2)
Zn—N3	2.095 (3)

**Table:** 

O1—Zn—O2	55.96 (12)
O1—Zn—O3	142.65 (12)
O1—Zn—O4	97.49 (10)
O1—Zn—N1	102.97 (9)
O1—Zn—N3	106.17 (10)
O2—Zn—O3	93.35 (10)
O2—Zn—O4	88.13 (10)
O2—Zn—N1	158.51 (9)
O2—Zn—N3	90.70 (9)
O3—Zn—O4	57.04 (10)
O3—Zn—N1	104.14 (10)
O3—Zn—N3	93.66 (11)
O4—Zn—N1	90.96 (10)
O4—Zn—N3	150.53 (10)
N1—Zn—N3	100.39 (10)

**Table 2:** Hydrogen-bond geometry (Å, °)

*D*—H⋯*A*	*D*—H	H⋯*A*	*D*⋯*A*	*D*—H⋯*A*
O7—H71⋯O1	0.97 (7)	2.08 (7)	2.910 (8)	144 (6)
O7—H72⋯O4^i^	0.92 (8)	1.81 (8)	2.713 (10)	167 (8)
N2—H2*A*⋯O5^ii^	0.86	2.07	2.905 (4)	165
N2—H2*B*⋯O6^iii^	0.86	2.15	2.977 (4)	161
N4—H4*A*⋯O2^iv^	0.86	2.14	2.961 (4)	159
N4—H4*B*⋯O2^v^	0.86	2.11	2.920 (4)	157

Symmetry codes: (i) 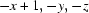; (ii) 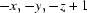; (iii) 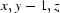; (iv) 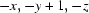; (v) 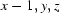.

**Fig. 1.**
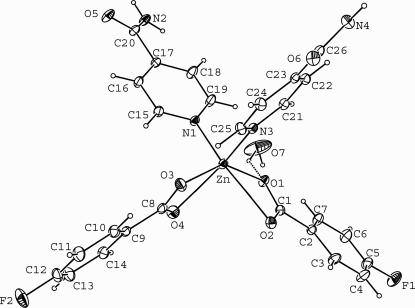
The molecular structure of the title compound. Displacement ellipsoids are drawn at the 10% probability level. Hydrogen bond is shown as dashed line.

**Fig. 2.**
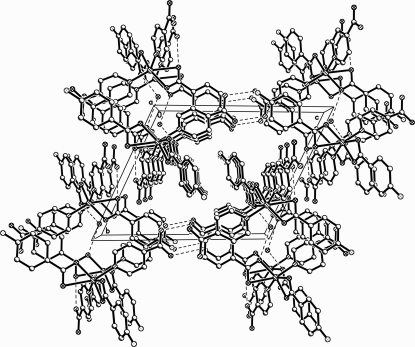
A packing diagram of the title compound, viewed down the a axis, showing hydrogen bonds (dashed lines) linking the molecules. H atoms have been omitted for clarity.

## supplementary crystallographic information

### Comment

Nicotinamide (NA) is one form of niacin. A deficiency of this vitamin leads to loss of copper from the body, known as pellagra disease. Victims of pellagra show unusually high serum and urinary copper levels (Krishnamachari, 1974). The nicotinic acid derivative *N*,*N*-diethylnicotinamide (DENA) is an important respiratory stimulant (Bigoli *et al.*, 1972). The structural functions and coordination relationships of the arylcarboxylate ion in zinc(II) complexes of benzoic acid derivatives may be changed, depending on the nature and position of the substituted groups on the benzene ring, the nature of the additional ligand molecule or solvent, and the medium of the synthesis (Adiwidjaja *et al.*, 1978; Antsyshkina *et al.*, 1980; Amiraslanov *et al.*, 1979; Nadzhafov, Shnulin & Mamedov, 1981; Shnulin *et al.*, 1981).

The solid-state structures of anhydrous zinc(II) carboxylates include one-dimensional (Clegg *et al.*, 1986*a*; Guseinov *et al.*, 1984), two-dimensional (Clegg *et al.*, 1986*b*, 1987) and three-dimensional (Capilla & Aranda, 1979) polymeric motifs of different types, while discerete monomeric complexes with octahedral or tetrahedral coordination geometry are found if water or other donor molecules are coordinated to Zn (Niekerk *et al.*, 1953; Usubaliev *et al.*, 1992). The structures of several complexes obtained by reacting Zn^II^ with NA and DENA have been determined in our laboratory, including those of [Zn(C_7_H_4_FO_2_)_2_(DENA)_2_(H_2_O)_2_], (II), (Hökelek *et al.*, 2007), [Zn(C_7_H_5_O_3_)_2_(NA)_2_], (III), (Necefoğlu *et al.*, 2002), [Zn(C_7_H_5_O_3_)(OH_2_)_3_(NA)].C_7_O_3_H_5_, (IV), (Hökelek & Necefoğlu, 2001), [Zn_2_(C_7_H_5_O_3_)_4_(DENA)_2_(H_2_O)_2_], (V), (Hökelek & Necefoğlu, 1996). In (III), one of the 4-hydroxybenzoate ions acts as bidentate ligand, while the other one is monodentate, but in (V), two of the benzoate ions act as monodentate ligands, while the other two are bidentate, bridging two Zn atoms. The structure determination of the title compound, (I), a zinc(II) complex with two fluorobenzoate (FB), two NA ligands and one uncoordinated water molecule, was undertaken in order to determine the properties of the FB and NA ligands and also to compare the results obtained with those reported previously.

In the monomeric title complex, the Zn^II^ atom is coordinated by two NA and two FB ligands. Two FB ions act as bidentate ligands, while two NA molecules are monodentate ligands (Fig. 1). Besides four short coordination bonds, the close contact of the O4 atom with the Zn atom [Zn···O4 = 2.458 (3) Å] may be considered to give the fifth coordination bond, as in (III) [Zn···O4 = 2.404 (2) Å]; this distance is much greater than the sum of the corresponding ionic radii (2.14 Å; Day & Selbin, 1969). Similar reported Zn···O contacts are 2.50 (1) Å in [Zn(*n*-HOC_6_H_4_COO)_2_(C_5_H_5_N)_2_].2C_5_H_5_N (Nadzhafov, Usubaliev *et al.*, 1981) and 2.494 (8) Å in [Zn(*p*-H_2_NC_6_H_4_COO)_2_]_n_.1.5nH_2_O (Amiraslanov *et al.*, 1980). On the other hand, the Zn···O2 distance [2.564 (3) Å] in (I) may also be considered as a coordination bond, although it is weak. Thus, the six-coordination geometry around the Zn^II^ atom may be described as highly distorted octahedral (Table 1), with the two nicotinamide ligands arranged *cis*.

In the binuclear complex (V), the average Zn—O bond length [1.953 (2) Å] is shorter than the corresponding value in (I) [2.253 (3) Å], but Zn is four-coordinate. In complexes (II), (III) and (IV), where Zn atoms are six-, five- and five-coordinate, the average Zn—O bond lengths are 2.117 (2) Å, 2.107 (2) Å and 2.047 (5) Å, respectively. The average Zn—N bond length [2.087 (3) Å] in (I) is in good agreement with the values reported in (III) [2.075 (2) Å], (IV) [2.089 (5) Å] and (V) [2.049 (2) Å], while it is shorter than the corresponding value in (II) [2.169 (3) Å]. The Zn atom lies -0.0682 (5) and -0.0030 (4) Å out of the O1/C1/O2 and O3/C8/O4 carboxyl planes, respectively.

In the carboxylate group, the C1—O1 and C8—O3 bond lengths [1.266 (4) and 1.264 (4) Å] are a little larger than the C1—O2 and C8—O4 [1.246 (4) and 1.229 (4) Å] bond lengths and may be compared with the corresponding distances: 1.260 (4) and 1.252 (4) Å in (II), 1.281 (3), 1.274 (3) and 1.240 (3), 1.245 (3) Å in (III) and 1.279 (4) and 1.246 (4) Å in (V). The dihedral angles between the mean planes of the carboxyl groups (O1/C1/O2 and O3/C8/O4) and the benzene rings [A (C2 to C7) and B (C9 to C14)] in the FB ligands are 8.2 (2) and 7.5 (2)°, respectively; these may be compared with the corresponding values of 2.8 (3)° in (II), 12.2 (2) and 10.0 (2)° in (III). The configuration around the Zn atom is given by the torsion angles. Rings A, B, C (N1/C15 to C19) and D (N3/C21 to C25) are, of course, planar and they are oriented with respect to each other at dihedral angles of A/B = 81.33 (13), A/C = 25.48 (13), A/D = 85.10 (12), B/C = 79.78 (12), B/D = 3.78 (11) and C/D = 83.06 (12)°.

As can be seen from the packing diagram (Fig. 2), the molecules of (I) are linked by intermolecular O—H···O and N—H···O hydrogen bonds (Table 2), forming a supramolecular structure.

### Experimental

The title compound was prepared by the reaction of Zn(NO_3_)_2_ (1.89 g, 10 mmol) in H_2_O (25 ml) and nicotinamide (2.16 g, 20 mmol) in H_2_O (25 ml) with sodium *p*-fluorobenzoate (3.24 g, 20 mmol) in H_2_O (100 ml). The mixture was filtered and set aside to crystallize at ambient temperature for several days, giving colorless single crystals.

### Refinement

H atoms of the water molecule were located in a difference Fourier map and refined with a fixed displacement parameter, *U*_iso_(H) = 0.237 Å^2^. The restrains on the O—H bond lengths and H—O—H bond angle of water molecule were applied. The remaining H atoms were positioned geometrically and refined as riding atoms, with N—H = 0.86 and C—H = 0.93 Å, and with *U*_iso_(H) = 1.2*U*_eq_(C,*N*).

### Crystal data

**Table:**

[Zn(C_7_H_4_FO_2_)_2_(C_6_H_6_N_2_O)_2_]·H_2_O	*Z* = 2
*M**_r_* = 605.87	*F*_000_ = 620
Triclinic, *P*1	*D*_x_ = 1.508 Mg m^−^^3^
Hall symbol: -P 1	Mo *K*α radiation λ = 0.71073 Å
*a* = 8.2363 (2) Å	Cell parameters from 25 reflections
*b* = 12.3711 (2) Å	θ = 6.3–15.8º
*c* = 14.8971 (3) Å	µ = 0.99 mm^−^^1^
α = 113.178 (14)º	*T* = 294 (2) K
β = 99.015 (17)º	Block, colorless
γ = 99.465 (16)º	0.30 × 0.25 × 0.20 mm
*V* = 1334.7 (2) Å^3^	

### Data collection

**Table:**

Enraf–Nonius TurboCAD-4 diffractometer	*R*_int_ = 0.058
Radiation source: fine-focus sealed tube	θ_max_ = 26.3º
Monochromator: graphite	θ_min_ = 2.6º
*T* = 294(2) K	*h* = 0→10
ω scans	*k* = −15→15
Absorption correction: ψ scan(North *et al.*, 1968)	*l* = −18→18
*T*_min_ = 0.735, *T*_max_ = 0.816	3 standard reflections
5794 measured reflections	every 120 min
5401 independent reflections	intensity decay: 1%
4454 reflections with *I* > 2σ(*I*)	

### Refinement

**Table:**

Refinement on *F*^2^	Secondary atom site location: difference Fourier map
Least-squares matrix: full	Hydrogen site location: inferred from neighbouring sites
*R*[*F*^2^ > 2σ(*F*^2^)] = 0.058	H atoms treated by a mixture of independent and constrained refinement
*wR*(*F*^2^) = 0.152	*w* = 1/[σ^2^(*F*_o_^2^) + (0.1013*P*)^2^ + 0.1012*P*] where *P* = (*F*_o_^2^ + 2*F*_c_^2^)/3
*S* = 1.14	(Δ/σ)_max_ < 0.001
5401 reflections	Δρ_max_ = 0.70 e Å^−^^3^
368 parameters	Δρ_min_ = −0.90 e Å^−^^3^
4 restraints	Extinction correction: SHELXL97 (Sheldrick, 2008), Fc^*^=kFc[1+0.001xFc^2^λ^3^/sin(2θ)]^-1/4^
Primary atom site location: structure-invariant direct methods	Extinction coefficient: 0.052 (4)

### Fractional atomic coordinates and isotropic or equivalent isotropic displacement parameters (Å^2^)

**Table:**

	*x*	*y*	*z*	*U*_iso_*/*U*_eq_	
Zn	0.39886 (4)	0.27400 (3)	0.12795 (2)	0.04482 (17)	
F1	0.3683 (5)	−0.0772 (3)	−0.46673 (18)	0.1135 (10)	
F2	1.3183 (4)	0.6557 (3)	0.5118 (2)	0.1174 (11)	
O1	0.3504 (3)	0.14478 (19)	−0.01064 (16)	0.0548 (5)	
O2	0.4950 (3)	0.30575 (19)	−0.01740 (17)	0.0551 (5)	
O3	0.5801 (3)	0.4167 (3)	0.2340 (2)	0.0702 (7)	
O4	0.6874 (4)	0.2602 (2)	0.1867 (2)	0.0769 (8)	
O5	0.1224 (4)	0.1149 (2)	0.48350 (18)	0.0745 (8)	
O6	−0.0625 (3)	0.71261 (19)	0.16243 (18)	0.0596 (6)	
O7	0.2133 (11)	−0.0996 (5)	−0.0339 (6)	0.230 (4)	
H71	0.295 (9)	−0.022 (4)	−0.005 (7)	0.237*	
H72	0.260 (10)	−0.154 (6)	−0.078 (6)	0.237*	
N1	0.3033 (3)	0.1816 (2)	0.20611 (18)	0.0459 (6)	
N2	0.0163 (4)	−0.0739 (3)	0.3616 (2)	0.0637 (8)	
H2A	−0.0266	−0.0998	0.4007	0.076*	
H2B	0.0043	−0.1228	0.2996	0.076*	
N3	0.2216 (3)	0.3771 (2)	0.12605 (18)	0.0447 (5)	
N4	−0.2859 (4)	0.5519 (3)	0.0811 (2)	0.0590 (7)	
H4A	−0.3543	0.5964	0.0773	0.071*	
H4B	−0.3235	0.4743	0.0560	0.071*	
C1	0.4207 (4)	0.1958 (3)	−0.0584 (2)	0.0456 (6)	
C2	0.4091 (4)	0.1219 (3)	−0.1675 (2)	0.0468 (7)	
C3	0.4631 (5)	0.1776 (3)	−0.2257 (3)	0.0686 (10)	
H3	0.5087	0.2611	−0.1964	0.082*	
C4	0.4501 (6)	0.1109 (4)	−0.3262 (3)	0.0845 (13)	
H4	0.4858	0.1482	−0.3655	0.101*	
C5	0.3844 (6)	−0.0099 (4)	−0.3665 (3)	0.0733 (10)	
C6	0.3290 (6)	−0.0687 (3)	−0.3127 (3)	0.0720 (10)	
H6	0.2827	−0.1522	−0.3432	0.086*	
C7	0.3433 (5)	−0.0015 (3)	−0.2115 (3)	0.0616 (9)	
H7	0.3081	−0.0400	−0.1729	0.074*	
C8	0.7031 (4)	0.3684 (3)	0.2396 (2)	0.0561 (8)	
C9	0.8671 (4)	0.4466 (3)	0.3131 (2)	0.0471 (7)	
C10	0.8903 (5)	0.5711 (3)	0.3645 (3)	0.0576 (8)	
H10	0.8025	0.6065	0.3537	0.069*	
C11	1.0424 (5)	0.6425 (4)	0.4315 (3)	0.0723 (11)	
H11	1.0592	0.7261	0.4657	0.087*	
C12	1.1677 (5)	0.5872 (4)	0.4462 (3)	0.0720 (10)	
C13	1.1497 (5)	0.4663 (4)	0.3980 (3)	0.0731 (11)	
H13	1.2379	0.4319	0.4103	0.088*	
C14	0.9975 (5)	0.3947 (3)	0.3299 (3)	0.0601 (8)	
H14	0.9831	0.3114	0.2954	0.072*	
C15	0.3619 (4)	0.2332 (3)	0.3051 (2)	0.0552 (8)	
H15	0.4493	0.3037	0.3344	0.066*	
C16	0.2988 (4)	0.1868 (3)	0.3668 (2)	0.0563 (8)	
H16	0.3419	0.2268	0.4362	0.068*	
C17	0.1730 (4)	0.0820 (3)	0.3253 (2)	0.0468 (7)	
C18	0.1122 (5)	0.0265 (3)	0.2222 (3)	0.0710 (11)	
H18	0.0274	−0.0455	0.1911	0.085*	
C19	0.1796 (5)	0.0799 (4)	0.1658 (3)	0.0714 (11)	
H19	0.1364	0.0430	0.0963	0.086*	
C20	0.0998 (5)	0.0398 (3)	0.3964 (2)	0.0536 (8)	
C21	0.0622 (4)	0.3283 (3)	0.0705 (2)	0.0474 (7)	
H21	0.0288	0.2448	0.0311	0.057*	
C22	−0.0549 (4)	0.3948 (3)	0.0684 (2)	0.0460 (6)	
H22	−0.1649	0.3563	0.0288	0.055*	
C23	−0.0092 (4)	0.5193 (2)	0.1254 (2)	0.0415 (6)	
C24	0.1563 (4)	0.5696 (3)	0.1836 (2)	0.0535 (7)	
H24	0.1932	0.6528	0.2237	0.064*	
C25	0.2657 (4)	0.4967 (3)	0.1819 (3)	0.0539 (7)	
H25	0.3760	0.5326	0.2217	0.065*	
C26	−0.1227 (4)	0.6028 (3)	0.1256 (2)	0.0451 (6)	

### Atomic displacement parameters (Å^2^)

**Table:**

	*U*^11^	*U*^22^	*U*^33^	*U*^12^	*U*^13^	*U*^23^
Zn	0.0522 (2)	0.0428 (2)	0.0383 (2)	0.00425 (15)	0.01085 (15)	0.01931 (16)
F1	0.165 (3)	0.109 (2)	0.0478 (13)	0.020 (2)	0.0416 (16)	0.0141 (14)
F2	0.0804 (17)	0.103 (2)	0.097 (2)	−0.0022 (15)	−0.0312 (15)	0.0000 (17)
O1	0.0764 (15)	0.0430 (11)	0.0404 (11)	0.0061 (10)	0.0155 (10)	0.0162 (9)
O2	0.0579 (13)	0.0396 (11)	0.0532 (13)	0.0014 (9)	0.0015 (10)	0.0138 (10)
O3	0.0574 (14)	0.0737 (17)	0.0754 (17)	0.0012 (12)	−0.0024 (12)	0.0409 (14)
O4	0.0813 (18)	0.0568 (15)	0.0639 (16)	−0.0109 (13)	0.0102 (14)	0.0110 (13)
O5	0.118 (2)	0.0515 (13)	0.0494 (14)	−0.0009 (14)	0.0378 (14)	0.0188 (11)
O6	0.0685 (14)	0.0359 (11)	0.0611 (14)	0.0063 (10)	0.0086 (11)	0.0128 (10)
O7	0.314 (9)	0.100 (4)	0.263 (8)	0.007 (5)	0.209 (7)	0.032 (4)
N1	0.0529 (14)	0.0467 (13)	0.0400 (12)	0.0058 (11)	0.0124 (11)	0.0229 (11)
N2	0.094 (2)	0.0480 (15)	0.0496 (16)	0.0031 (15)	0.0304 (15)	0.0221 (13)
N3	0.0516 (13)	0.0441 (13)	0.0379 (12)	0.0068 (11)	0.0113 (10)	0.0190 (10)
N4	0.0539 (15)	0.0421 (13)	0.078 (2)	0.0074 (12)	0.0060 (14)	0.0285 (14)
C1	0.0475 (15)	0.0436 (15)	0.0431 (15)	0.0108 (12)	0.0083 (12)	0.0172 (12)
C2	0.0505 (16)	0.0437 (15)	0.0422 (15)	0.0080 (12)	0.0104 (12)	0.0164 (12)
C3	0.087 (3)	0.055 (2)	0.059 (2)	−0.0021 (18)	0.0273 (19)	0.0243 (17)
C4	0.110 (3)	0.086 (3)	0.063 (2)	0.008 (3)	0.039 (2)	0.037 (2)
C5	0.088 (3)	0.082 (3)	0.0430 (18)	0.020 (2)	0.0239 (18)	0.0166 (18)
C6	0.103 (3)	0.0466 (18)	0.0481 (19)	0.0094 (19)	0.0173 (19)	0.0058 (15)
C7	0.086 (2)	0.0482 (17)	0.0474 (18)	0.0106 (17)	0.0178 (17)	0.0196 (14)
C8	0.0600 (19)	0.065 (2)	0.0458 (17)	−0.0015 (16)	0.0115 (14)	0.0338 (16)
C9	0.0522 (16)	0.0520 (16)	0.0373 (14)	0.0053 (13)	0.0119 (12)	0.0219 (13)
C10	0.0627 (19)	0.0558 (18)	0.0514 (18)	0.0169 (15)	0.0130 (15)	0.0196 (15)
C11	0.082 (3)	0.051 (2)	0.056 (2)	0.0103 (18)	0.0054 (19)	0.0022 (16)
C12	0.059 (2)	0.074 (2)	0.051 (2)	0.0022 (18)	−0.0064 (16)	0.0081 (18)
C13	0.062 (2)	0.077 (3)	0.071 (2)	0.0246 (19)	0.0039 (19)	0.024 (2)
C14	0.069 (2)	0.0504 (18)	0.0531 (19)	0.0128 (16)	0.0121 (16)	0.0161 (15)
C15	0.0567 (18)	0.0554 (18)	0.0450 (16)	−0.0059 (15)	0.0072 (14)	0.0228 (14)
C16	0.0651 (19)	0.0599 (19)	0.0378 (15)	−0.0006 (15)	0.0093 (14)	0.0225 (14)
C17	0.0584 (17)	0.0448 (15)	0.0439 (15)	0.0130 (13)	0.0184 (13)	0.0235 (13)
C18	0.092 (3)	0.058 (2)	0.0443 (18)	−0.0178 (19)	0.0138 (18)	0.0188 (16)
C19	0.088 (3)	0.072 (2)	0.0381 (16)	−0.016 (2)	0.0103 (17)	0.0226 (16)
C20	0.072 (2)	0.0472 (16)	0.0471 (17)	0.0085 (15)	0.0245 (15)	0.0242 (14)
C21	0.0565 (17)	0.0362 (14)	0.0408 (14)	0.0014 (12)	0.0078 (13)	0.0133 (12)
C22	0.0476 (15)	0.0407 (14)	0.0402 (14)	−0.0001 (12)	0.0045 (12)	0.0145 (12)
C23	0.0504 (15)	0.0396 (14)	0.0349 (13)	0.0036 (12)	0.0121 (11)	0.0188 (11)
C24	0.0565 (18)	0.0373 (14)	0.0522 (17)	−0.0011 (13)	0.0031 (14)	0.0134 (13)
C25	0.0503 (17)	0.0437 (16)	0.0545 (18)	0.0015 (13)	0.0016 (14)	0.0159 (14)
C26	0.0553 (17)	0.0424 (15)	0.0373 (14)	0.0059 (13)	0.0117 (12)	0.0191 (12)

### Geometric parameters (Å, °)

**Table:**

Zn—O1	1.978 (2)	C6—H6	0.9300
Zn—O2	2.564 (3)	C7—C6	1.379 (5)
Zn—O3	2.010 (3)	C7—H7	0.9300
Zn—O4	2.458 (3)	C9—C14	1.377 (5)
Zn—N1	2.079 (2)	C9—C10	1.386 (4)
Zn—N3	2.095 (3)	C9—C8	1.494 (4)
F1—C5	1.365 (4)	C10—H10	0.9300
F2—C12	1.354 (4)	C11—C10	1.376 (5)
O2—C1	1.246 (4)	C11—C12	1.364 (6)
O3—C8	1.264 (4)	C11—H11	0.9300
O4—C8	1.229 (4)	C12—C13	1.350 (6)
O5—C20	1.225 (4)	C13—H13	0.9300
O6—C26	1.224 (4)	C14—C13	1.384 (5)
O7—H71	0.97 (8)	C14—H14	0.9300
O7—H72	0.92 (8)	C15—C16	1.383 (4)
N1—C15	1.322 (4)	C15—H15	0.9300
N1—C19	1.332 (4)	C16—H16	0.9300
N2—C20	1.310 (4)	C17—C16	1.365 (4)
N2—H2A	0.8600	C17—C18	1.377 (4)
N2—H2B	0.8600	C17—C20	1.514 (4)
N3—C25	1.332 (4)	C18—H18	0.9300
N3—C21	1.333 (4)	C19—C18	1.382 (5)
N4—H4A	0.8600	C19—H19	0.9300
N4—H4B	0.8600	C21—H21	0.9300
C1—O1	1.266 (4)	C22—C21	1.372 (4)
C1—C2	1.497 (4)	C22—H22	0.9300
C2—C3	1.384 (4)	C23—C22	1.384 (4)
C2—C7	1.372 (4)	C23—C24	1.387 (4)
C3—C4	1.371 (6)	C23—C26	1.502 (4)
C3—H3	0.9300	C24—C25	1.371 (5)
C4—H4	0.9300	C24—H24	0.9300
C5—C4	1.346 (6)	C25—H25	0.9300
C5—C6	1.359 (6)	C26—N4	1.326 (4)
			
O1—Zn—O2	55.96 (12)	O4—C8—Zn	70.8 (2)
O1—Zn—O3	142.65 (12)	C9—C8—Zn	168.1 (3)
O1—Zn—O4	97.49 (10)	C10—C9—C8	120.9 (3)
O1—Zn—N1	102.97 (9)	C14—C9—C10	119.4 (3)
O1—Zn—N3	106.17 (10)	C14—C9—C8	119.7 (3)
O2—Zn—O3	93.35 (10)	C9—C10—H10	119.8
O2—Zn—O4	88.13 (10)	C11—C10—C9	120.4 (3)
O2—Zn—N1	158.51 (9)	C11—C10—H10	119.8
O2—Zn—N3	90.70 (9)	C10—C11—H11	120.9
O3—Zn—O4	57.04 (10)	C12—C11—C10	118.2 (3)
O3—Zn—N1	104.14 (10)	C12—C11—H11	120.9
O3—Zn—N3	93.66 (11)	F2—C12—C11	119.1 (4)
O4—Zn—N1	90.96 (10)	C13—C12—F2	117.7 (4)
O4—Zn—N3	150.53 (10)	C13—C12—C11	123.1 (3)
N1—Zn—N3	100.39 (10)	C12—C13—C14	118.7 (4)
C1—O1—Zn	104.58 (18)	C12—C13—H13	120.7
C8—O3—Zn	101.0 (2)	C14—C13—H13	120.7
C8—O4—Zn	81.0 (2)	C9—C14—C13	120.1 (3)
H72—O7—H71	107 (4)	C9—C14—H14	119.9
C15—N1—C19	117.5 (3)	C13—C14—H14	119.9
C15—N1—Zn	116.9 (2)	N1—C15—C16	122.8 (3)
C19—N1—Zn	125.4 (2)	N1—C15—H15	118.6
C20—N2—H2A	120.0	C16—C15—H15	118.6
C20—N2—H2B	120.0	C15—C16—H16	120.2
H2A—N2—H2B	120.0	C17—C16—C15	119.6 (3)
C21—N3—Zn	122.8 (2)	C17—C16—H16	120.2
C25—N3—C21	117.0 (3)	C16—C17—C18	118.1 (3)
C25—N3—Zn	120.2 (2)	C16—C17—C20	117.5 (3)
C26—N4—H4A	120.0	C18—C17—C20	124.2 (3)
C26—N4—H4B	120.0	C17—C18—C19	118.8 (3)
H4A—N4—H4B	120.0	C17—C18—H18	120.6
O1—C1—C2	118.5 (3)	C19—C18—H18	120.6
O2—C1—O1	121.6 (3)	N1—C19—C18	123.2 (3)
O2—C1—C2	119.8 (3)	N1—C19—H19	118.4
C3—C2—C1	120.2 (3)	C18—C19—H19	118.4
C7—C2—C3	119.1 (3)	O5—C20—N2	124.0 (3)
C7—C2—C1	120.7 (3)	O5—C20—C17	117.5 (3)
C2—C3—H3	119.7	N2—C20—C17	118.5 (3)
C4—C3—C2	120.7 (3)	N3—C21—C22	123.4 (3)
C4—C3—H3	119.7	N3—C21—H21	118.3
C3—C4—H4	120.8	C22—C21—H21	118.3
C5—C4—C3	118.4 (4)	C21—C22—C23	119.9 (3)
C5—C4—H4	120.8	C21—C22—H22	120.0
C4—C5—C6	123.2 (3)	C23—C22—H22	120.0
C4—C5—F1	119.1 (4)	C22—C23—C24	116.5 (3)
C6—C5—F1	117.8 (4)	C22—C23—C26	125.0 (3)
C5—C6—C7	118.3 (3)	C24—C23—C26	118.5 (3)
C5—C6—H6	120.9	C23—C24—H24	120.0
C7—C6—H6	120.9	C25—C24—C23	120.1 (3)
C2—C7—C6	120.4 (3)	C25—C24—H24	120.0
C2—C7—H7	119.8	N3—C25—C24	123.2 (3)
C6—C7—H7	119.8	N3—C25—H25	118.4
O3—C8—Zn	50.10 (17)	C24—C25—H25	118.4
O3—C8—C9	118.0 (3)	O6—C26—N4	122.7 (3)
O4—C8—O3	120.9 (3)	O6—C26—C23	120.1 (3)
O4—C8—C9	121.1 (3)	N4—C26—C23	117.2 (3)
			
O1—Zn—N1—C15	159.4 (2)	C3—C2—C7—C6	−0.9 (6)
O1—Zn—N1—C19	−25.8 (3)	C1—C2—C7—C6	178.4 (4)
O3—Zn—N1—C15	5.4 (3)	C7—C2—C3—C4	0.5 (6)
O3—Zn—N1—C19	−179.9 (3)	C1—C2—C3—C4	−178.8 (4)
O4—Zn—N1—C15	61.5 (3)	C6—C5—C4—C3	0.6 (8)
O4—Zn—N1—C19	−123.7 (3)	F1—C5—C4—C3	179.0 (4)
N3—Zn—N1—C15	−91.1 (3)	C2—C3—C4—C5	−0.3 (7)
N3—Zn—N1—C19	83.6 (3)	C4—C5—C6—C7	−1.0 (7)
O1—Zn—N3—C21	35.9 (2)	F1—C5—C6—C7	−179.4 (4)
O1—Zn—N3—C25	−144.1 (2)	C2—C7—C6—C5	1.1 (6)
O3—Zn—N3—C21	−176.1 (2)	C14—C9—C8—O4	−6.9 (4)
O3—Zn—N3—C25	3.9 (2)	C10—C9—C8—O4	172.9 (3)
O4—Zn—N3—C21	178.0 (2)	C14—C9—C8—O3	172.5 (3)
O4—Zn—N3—C25	−2.0 (3)	C10—C9—C8—O3	−7.7 (4)
N1—Zn—N3—C21	−71.0 (2)	C14—C9—C8—Zn	170.6 (8)
N1—Zn—N3—C25	109.0 (2)	C10—C9—C8—Zn	−9.6 (12)
O3—Zn—O1—C1	−38.7 (3)	C14—C9—C10—C11	0.3 (5)
O4—Zn—O1—C1	−81.6 (2)	C8—C9—C10—C11	−179.5 (3)
N1—Zn—O1—C1	−174.32 (19)	C10—C9—C14—C13	0.4 (5)
N3—Zn—O1—C1	80.6 (2)	C8—C9—C14—C13	−179.8 (3)
O1—Zn—O3—C8	−53.5 (3)	C12—C11—C10—C9	−0.7 (6)
N1—Zn—O3—C8	81.8 (2)	C10—C11—C12—C13	0.4 (7)
N3—Zn—O3—C8	−176.5 (2)	C10—C11—C12—F2	180.0 (4)
O4—Zn—O3—C8	0.04 (18)	F2—C12—C13—C14	−179.3 (4)
O1—Zn—O4—C8	150.46 (19)	C11—C12—C13—C14	0.3 (7)
O3—Zn—O4—C8	−0.04 (19)	C9—C14—C13—C12	−0.7 (6)
N1—Zn—O4—C8	−106.3 (2)	N1—C15—C16—C17	1.3 (6)
N3—Zn—O4—C8	7.0 (3)	C18—C17—C16—C15	−0.5 (5)
Zn—O3—C8—O4	−0.1 (4)	C20—C17—C16—C15	−175.8 (3)
Zn—O3—C8—C9	−179.5 (2)	C16—C17—C18—C19	−0.7 (6)
Zn—O4—C8—O3	0.1 (3)	C20—C17—C18—C19	174.2 (4)
Zn—O4—C8—C9	179.5 (3)	C16—C17—C20—O5	19.1 (5)
C19—N1—C15—C16	−0.8 (6)	C18—C17—C20—O5	−155.9 (4)
Zn—N1—C15—C16	174.4 (3)	C16—C17—C20—N2	−161.1 (3)
C15—N1—C19—C18	−0.5 (6)	C18—C17—C20—N2	23.9 (5)
Zn—N1—C19—C18	−175.2 (3)	N1—C19—C18—C17	1.3 (7)
C25—N3—C21—C22	0.2 (4)	C23—C22—C21—N3	0.6 (5)
Zn—N3—C21—C22	−179.8 (2)	C24—C23—C22—C21	−0.9 (4)
C21—N3—C25—C24	−0.5 (5)	C26—C23—C22—C21	176.7 (3)
Zn—N3—C25—C24	179.5 (3)	C22—C23—C24—C25	0.6 (5)
O2—C1—O1—Zn	−2.1 (3)	C26—C23—C24—C25	−177.2 (3)
C2—C1—O1—Zn	179.6 (2)	C23—C24—C25—N3	0.1 (5)
O2—C1—C2—C7	173.3 (3)	C22—C23—C26—O6	−165.7 (3)
O1—C1—C2—C7	−8.3 (5)	C24—C23—C26—O6	11.9 (4)
O2—C1—C2—C3	−7.4 (5)	C22—C23—C26—N4	11.7 (4)
O1—C1—C2—C3	171.0 (3)	C24—C23—C26—N4	−170.7 (3)

### Hydrogen-bond geometry (Å, °)

**Table:**

*D*—H···*A*	*D*—H	H···*A*	*D*···*A*	*D*—H···*A*
O7—H71···O1	0.97 (7)	2.08 (7)	2.910 (8)	144 (6)
O7—H72···O4^i^	0.92 (8)	1.81 (8)	2.713 (10)	167 (8)
N2—H2A···O5^ii^	0.86	2.07	2.905 (4)	165
N2—H2B···O6^iii^	0.86	2.15	2.977 (4)	161
N4—H4A···O2^iv^	0.86	2.14	2.961 (4)	159
N4—H4B···O2^v^	0.86	2.11	2.920 (4)	157

Symmetry codes: (i) −*x*+1, −*y*, −*z*; (ii) −*x*, −*y*, −*z*+1; (iii) *x*, *y*−1, *z*; (iv) −*x*, −*y*+1, −*z*; (v) *x*−1, *y*, *z*.
